# Integrative taxonomy and whole genome sequencing of two new isopod species of the genus *Notopais* Hodgson, 1910 (Crustacea, Malacostraca) from the Southern Ocean

**DOI:** 10.1007/s12526-026-01727-0

**Published:** 2026-11-12

**Authors:** Davide Di Franco, Maria Nilsson, Tilman Schell, Huw J. Griffiths, Katrin Linse, Katharina Krüger, Andreas Kelch, Angelika Brandt

**Affiliations:** 1 Department of Marine Zoology, Senckenberg Research Institute and Natural History Museum, Frankfurt Am Main, 60325 Germany; 2 GeoSphere Austria, Paleontology and Collections, Neulinggasse 38, Vienna, 1030 Austria; 3 Institute for Ecology, Diversity and Evolution, Goethe University Frankfurt, Frankfurt Am Main, 60438 Germany; 4 British Antarctic Survey; High Cross, Madingley Road, Cambridge, CB3 0ET UK; 5 Senckenberg Biodiversity and Climate Research Centre, Senckenberganlage 25, Frankfurt Am Main, 60325 Germany; 6 LOEWE Centre for Translational Biodiversity Genomics (LOEWE-TBG), Senckenberganlage 25, Frankfurt Am Main, 60325 Germany

**Keywords:** Antarctic, Analyses, Barcode, Genomic, Munnopsidae

## Abstract

The genus *Notopais* Hodgson, 1910 is distributed in the Southern Hemisphere and comprises a total of 11 species. In the present study, we describe two new species of the genus, *Notopais cryophila* sp. nov. and *Notopais polaris* sp. nov. using integrative taxonomy. The two species can be easily identified and distinguished from the other *Notopais* species by the spinal and tubercular pattern on the dorsal surface of their pereonites. The morphological distinction between the two new *Notopais* species was confirmed by a COI barcoding analysis, as well as by their different genome size of 1.44 Gb and 2.59 Gb and large differences in genomic heterozygosity (1.52%/0.70%). In the present work, we provide the first whole genome sequencing of isopod species from the Southern Ocean and the first mitochondrial genome of the genus *Notopais*.

**Supplementary Information:**

The online version contains supplementary material available at https://doi.org/10.1007/s12526-026-01727-0.

## Introduction

The family Munnopsidae Lilljeborg, 1864 is the most abundant and diverse group of deep-sea isopods. It comprises nine subfamilies and 41 genera, with more than 500 described species (WoRMS Editorial Board 2026), and it is frequently sampled in large numbers in the Southern Ocean (SO) (Brandt et al. 2004; Malyutina and Brandt 2007). Among munnopsid genera, the genus *Notopais* has only been recorded in the Southern Hemisphere (Merrin 2004) with a total number of 11 formally described species (WoRMS Editorial Board 2026). Outside the SO, several *Notopais* species have been described from temperate regions, including *Notopais echinatus* Merrin & Bruce, 2006 from the East Central Australian shelf, *Notopais euaxos* Merrin & Bruce, 2006 from the Chatham Rise, New Zealand, *Notopais minya* Merrin, 2004 from the south-eastern Australian continental slope, *Notopais zealandica* Merrin, 2004 from the south-eastern coast of New Zealand; more recently, *Notopais chathamensis* Merrin, 2024 was described from New Zealand waters, and *Notopais likros* Merrin, 2024 from off the east coast of Australia (Merrin, 2024). From the SO, to date the total number of formally described species known is five, comprising *Notopais beddardi* Merrin, 2004, *Notopais magnifica* (Vanhöffen, 1914), *Notopais quadrispinosa* (Beddard, 1886); *Notopais spicatus* Hodgson, 1910, and *Notopais spinosa* (Hodgson, 1902). Further *Notopais* specimens unassigned to known species are present in collections. New genetic methodologies (e.g., DNA barcoding) revealed to be a very useful approach for the identification of species and a valid complement to the classic taxonomy based on morphological characters (Bucklin et al. 2011; Leray and Knowlton 2015). However, genetic analyses on SO munnopsids are not numerous due to the difficulties in obtaining high-quality DNA from the isopod specimens, as was shown previously (Riehl et al. 2014). The most comprehensive study on munnopsids based on genetic data was performed in 2009 by Osborn; the author investigated the phylogenetic relationship within the family Munnopsidae using three different genetic markers; however, the study included only two of the described *Notopais* species (Osborn 2009). Beyond single-marker approaches such as DNA barcoding, the use of nuclear and mitochondrial genomic data is increasingly recognized as a powerful tool in taxonomy and phylogenetics (DeSalle and Goldstein 2019; Köhler et al. 2021; Letsch et al. 2025). Genome-wide data provide substantially more information than individual mitochondrial markers, allowing the assessment of genome size, heterozygosity and overall genetic divergence, as well as offering independent nuclear and mitochondrial perspectives on species boundaries and evolutionary history (Köhler et al. 2021; Letsch et al. 2025). In addition, complete mitochondrial genomes can overcome limitations associated with primer specificity and variable evolutionary rates of commonly used barcoding markers such as COI, which have been shown to perform inconsistently across marine invertebrate taxa (Riehl et al. 2014; Rach et al. 2017). Recent studies have demonstrated that mitochondrial genomes provide valuable phylogenetic signals for isopods and other crustaceans, supporting their use in systematic and evolutionary studies (Kilpert and Podsiadlowski 2006; Shen et al. 2017; Yu et al. 2018). Despite these advantages, genomic approaches are still rarely applied in isopod systematics, particularly for deep-sea and Southern Ocean taxa, mainly due to difficulties in obtaining suitable material and high-quality DNA (Raupach et al. 2004; Brix et al. 2011; Riehl et al. 2014; Thorpe 2024). Consequently, the integration of genomic data into taxonomic studies of isopods remains in its infancy. The aim of our study is to describe two new species of the genus *Notopais* based upon morphological description and DNA barcoding, to expand the knowledge of the distribution of *Notopais* species in the SO. In addition, we provide data from the nuclear and mitochondrial genome of the two new species. This is the first study reporting the complete nuclear genome of isopods from the Southern Ocean and of the genus *Notopais*.

## Material and methods

### Sampling

**Fig. 1:**
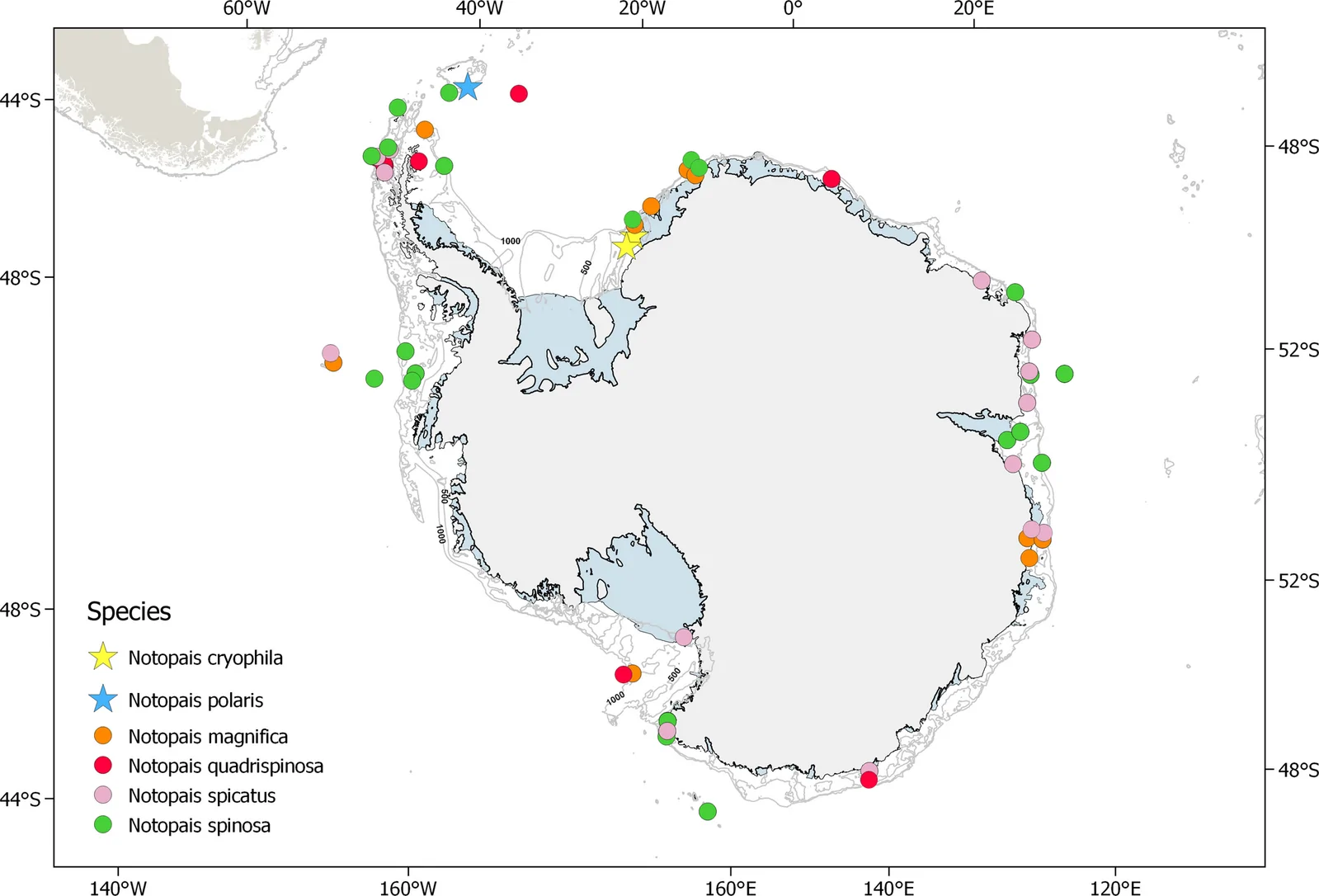
Map of *Notopais* species’ distribution in the Southern Ocean, based on GBIF and OBIS databases

Samples were collected during the British Antarctic Survey (BAS) expeditions JR275 and JR15005 on board of the research vessel RRS *James Clark Ross*, in the Filchner Trough area, Weddell Sea (2002), and in the South Orkney Islands (2016) respectively (Griffiths 2002, 2017) (Fig. 1). *Notopais* specimens were collected using an epibenthic sledge (EBS) (Brenke 2005) and stored into precooled (−20 °C) 96% ethanol. For later genetic studies, samples were retained in a −20 °C freezer for 48 h to reduce DNA degradation. Afterwards, they were stored at room temperature.

### Identification and species description

The identification of the new species, taxonomic drawings and comparison with the other SO species of the same genus were performed using two stereomicroscopes, Leica M60 and Leica MZ8, both equipped with a camera lucida. Appendage drawings were prepared by means of an equally equipped Leica DM750 microscope. In total, ten specimens (Table S1) were used for the illustrations. The allotype and paratype of the two new species of *Notopais*, as well as specimens used for morphological comparison and identified as *N. magnifica* (Vanhöffen, 1914), *N. spicatus* Hodgson, 1910, *N. spinosa* (Hodgson, 1902), and *N. quadrispinosa* (Beddard, 1886), were drawn in glycerine, whereas the holotypes of the two new species were drawn in 96% ethanol. For the glycerine drawings, specimens were transferred into a 1:1 solution of glycerine and 96% ethanol. After the ethanol volatilized completely, appendages from allo- and paratypes were dissected and transferred onto slides with pure glycerine. Once line drawings were completed, they were digitalized using a Wacom Intuos drawing pad and Adobe Illustrator CS2, following Coleman (2003). Holotypes were used to make the body descriptions, while the appendages were described from the allo- and paratypes (Table S1). Subsequently, the width of pereonites 1–4 was measured from spine to spine, as well as the total body length from the frontal edge of the cephalon to the caudal tip of the pleotelson. The remaining measurements were taken following Hessler (Hessler 1970).

The type material of the new, formally described *Notopais* species is held at the Senckenberg Research Institute and Natural History Museum in Frankfurt am Main, Germany.

### Comparative plate and biogeographic distribution

**Fig. 2:**
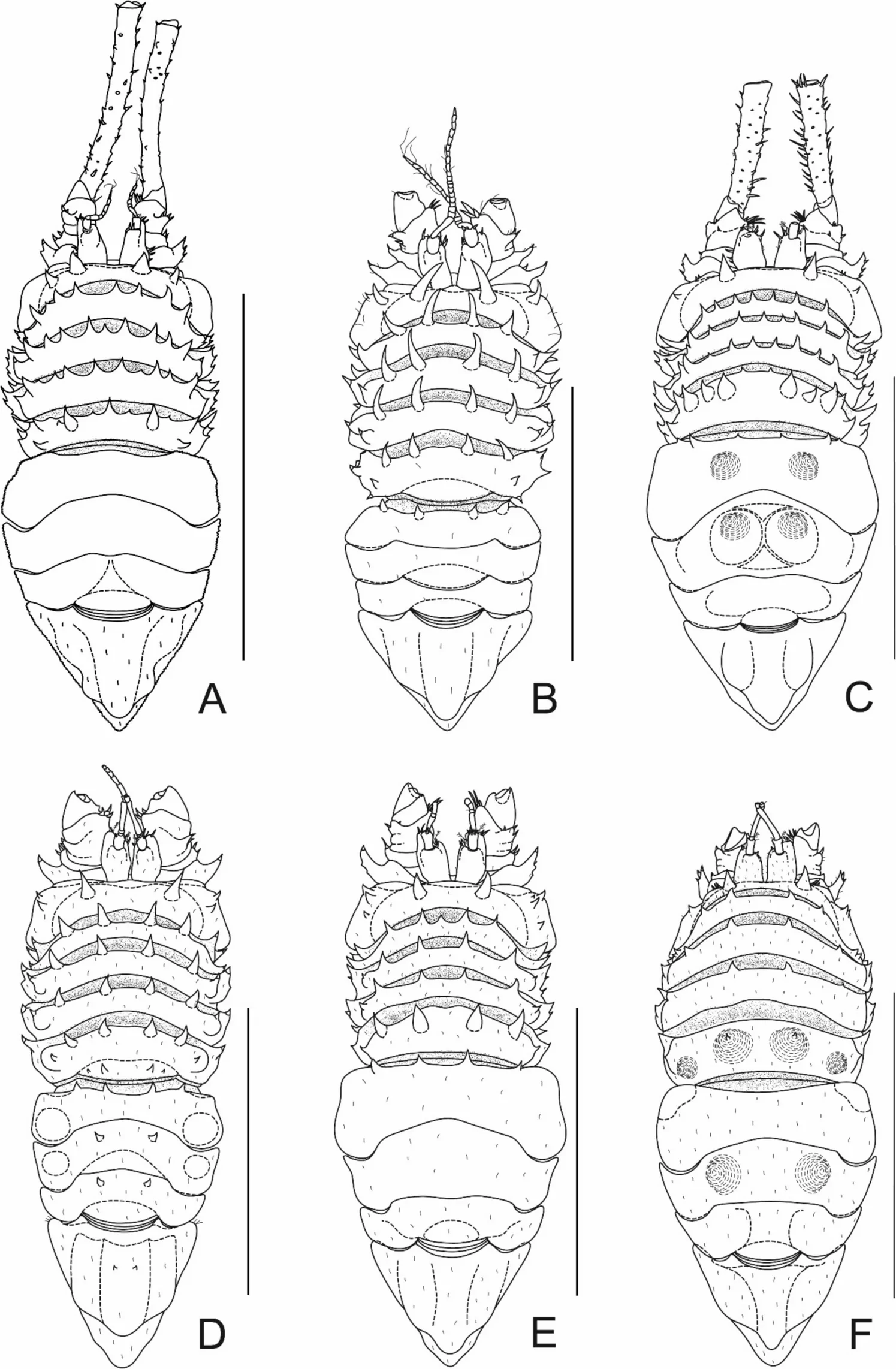
Southern Ocean species of *Notopais* Hodgson, 1910, dorsal view. **A** *N. magnifica* (Vanhöffen, 1914) (SMF-58534); **B** *N. spicatus* Hodgson, 1910 (SMF-58535); **C** *N. polaris* sp. nov. (SMF-58531); **D** *N. spinosa* (Hodgson, 1902) (SMF-58536); **E** *N. quadrispinosa* (Beddard, 1886) (SMF-58537); **F** *N. cryophila* sp. nov. (SMF-58528). Scale bars: **A**, **C**–**F** = 3 mm; **B** = 2 mm

**Fig. 3:**
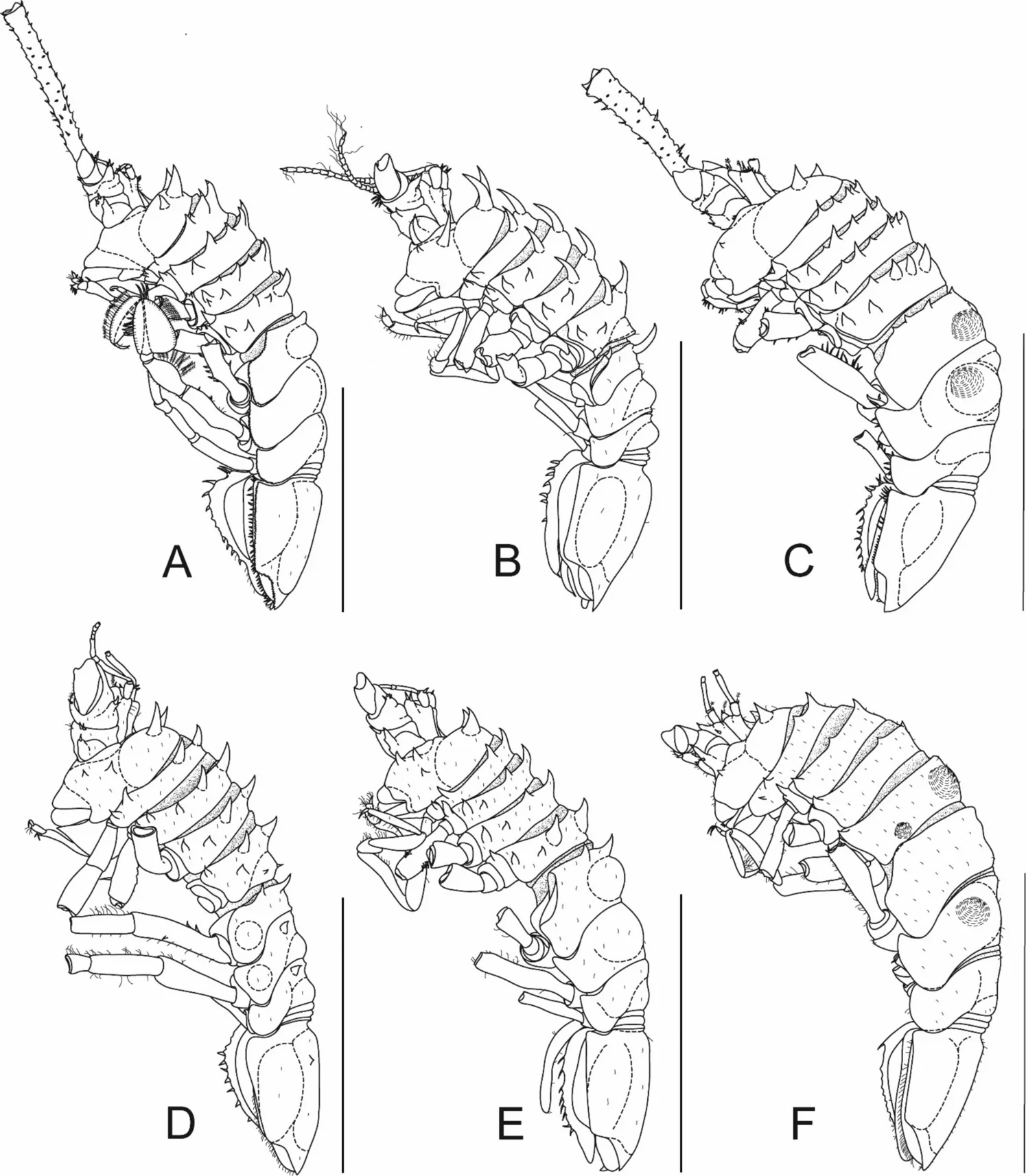
Southern Ocean species of *Notopais* Hodgson, 1910, lateral view. **A** *N. magnifica* (Vanhöffen, 1914) (SMF-58534); **B** *N. spicatus* Hodgson, 1910 (SMF-58535); **C** *N. polaris* sp. nov. (SMF-58531); **D** *N. spinosa* (Hodgson, 1902) (SMF-58536); **E** *N. quadrispinosa* (Beddard, 1886) (SMF-58537); **F** *N. cryophila* sp. nov. (SMF-58528). Scale bars: **A**, **B** = 2 mm; **C**–**F** = 3 mm

Specimens identified as *Notopais magnifica* (Vanhöffen, 1914), *Notopais spicatus* Hodgson, 1910, *Notopais spinosa* (Hodgson, 1902), and *Notopais quadrispinosa* (Beddard, 1886) were used for the preparation of the comparative plates (Figs. 2 and 3; Supplementary Table 1). These specimens were collected using an EBS on board RRS *James Clark Ross* during the expeditions JR275, JR15005 and JR 17003 a (Linse 2018) performed in the Filchner Trough, the South Orkney Islands and the Prince Gustav Channel respectively. Coordinates and depths of the sampling locations are shown in Table 1.

**Table 1:** Sampling coordinates and depth ranges of the *Notopais* specimens used for the comparative plates

Species	Start coordinates	End coordinates	Depth (m)
*Notopais magnifica* (Vanhöffen, 1914)	60°28.53′S, 44°25.38′W	60°28.41′S, 44°25.61′W	788–817
*Notopais spicatus* Hodgson, 1910	76°10.01′S, 27°48.23′W	76°09.94′S, 27°48.44′W	539–549
*Notopais spinosa* (Hodgson, 1902)	63°48.44′S, 58°04.12′W	63°48.57′S, 58°04.34′W	872–874
*Notopais quadrispinosa* (Beddard, 1886)	75°14.46′S, 27°51.72′W	75°14.55′S, 27°52.26′W	413–416

The distribution of the genus *Notopais* in the Southern Ocean shown in Fig. 1 is based on data retrieved from the databases GBIF (Global Biodiversity Information Facility) (https://www.gbif.org/) (GBIF.Org User 2021) and OBIS (Ocean Biodiversity Information System) (https://obis.org/) (Grassle 2000).

### Genetic analysis

Genetic analyses were performed on one paratype per species in order to preserve the integrity of the holotypes. The whole specimens had to be used for DNA isolation in order to obtain sufficient amounts of DNA. The paratype of the first new species was an adult female from the Filchner Trough area, sampled between 77°21.42′S–35°21.64′W and 77°21.47′S–35°21.90′W at a depth range of 654–656 m respectively; the paratype of the second new species was a manca (stage III) sampled in the South Orkney Islands between 62° 09.61′S–44°58.92′W and 62°09.45′S–44° 59.00′W (561–524 m respectively). The sex of this latter paratype could not be determined since the pleotelson was missing.

DNA was extracted from the two paratypes using an adaptation of the protocol by Miller et al. (1988) for high molecular weight DNA extraction. The DNA samples were used for short read Illumina genome sequencing. One library with an insert size of 350 basepairs (bp) was prepared per species and 150 bp paired-end reads were sequenced on the Illumina NovaSeq 6000 platform (San Diego, CA). The short read data from both samples was analyzed the same way. A k-mer profile was generated from the raw reads using Jellyfish 2.3.0 (Marçais and Kingsford 2011) and analyzed in the GenomeScope 1.0.0 (Vurture et al. 2017). Raw reads were trimmed for low-quality regions and adapter sequences and filtered for possible contamination using Trimmomatic 0.39 (Bolger et al. 2014) and Kraken 2.0.9 (Wood et al. 2019) respectively. Optimal k-mer length for de novo genome assembly was estimated using KmerGenie 1.7051 (Chikhi and Medvedev 2014). Subsequently the unclassified paired and unpaired reads were assembled using Velvet 1.2.10 (Zerbino and Birney 2008). Basic assembly statistics were determined using Quast 5.0.2 (Gurevich et al. 2013). Backmapping (a procedure where the reads used for the assembly are mapped again to the assembly) was performed with backmap.pl 0.3 (https://github.com/schellt/backmap), which utilized bwa mem 0.7.17-r1188 (Li 2013), samtools 1.10 (Li et al. 2009), Qualimap 2.2.1 (Okonechnikov et al. 2016), bedtools 2.28.0 (Quinlan and Hall 2010), R 4.0.3 (R Core Team 2020), and MultiQC 1.9 (Ewels et al. 2016). Possible contamination was identified with Blobtools 1.1.1 (Laetsch and Blaxter 2017) and screening for single-copy orthologs with BUSCO 4.1.4 (Simão et al. 2015) using the arthropoda_odb10 data set. From the initial assembly contigs originating from contamination (either taxonomic assignment to bacteria, virus, and/or coverage < 10 and GC > 0.5) or the mitochondrial genome were filtered out. Afterwards, the quality checks (BUSCO, backmap, blobtools) were repeated as described above.

The mitochondrial genomes were assembled using first, MitoZ 2.3 on a 3 Gb subset of unclassified read pairs that stayed at 150 bp full length after trimming each. Since these assemblies were insufficient, the longest sequence of a protein coding gene from MitoZ was used as seed in NOVOPlasty 4.2 (Dierckxsens et al. 2016). Annotations of the mitochondrial genomes were manually merged and curated in Geneious Prime 2021.1.1 (https://www.geneious.com) using automated annotations from MITOS2 (Donath et al. 2019) and GeSeq (Tillich et al. 2017).

The CO1 gene sequences were extracted from the complete mitochondrial genome from the two new *Notopais* species and aligned to sequences of selected Munnopsidae isopods available in GenBank (Table 2). Species were selected to represent different genera within the family in order to assess the phylogenetic placement of the new taxa within Munnopsidae. A published sequence from the genus *Santia* in the family Santiidae was chosen as the outgroup taxon (Lindquist et al. 2005), as both families sit within the Superfamily Janiroidea. Sequences were aligned with MUSCLE (Edgar 2004) as implemented in the software AliView 1.26 (Larsson 2014) and manually inspected. A phylogenetic analysis was done using maximum likelihood (ML) using the CO1 sequence alignment with IQ-TREE 1.6.12 (http://www.iqtree.org/) (Minh et al. 2013; Nguyen et al. 2015; Trifinopoulos et al. 2016). Ultrafast bootstrap analysis was used to assess for node support (Hoang et al. 2018), this latter was tested using SH-aLRT branch test based on 1000 replicates. The phylogenetic tree was visualized using FigTree 1.4.4 (available at http://tree.bio.ed.ac.uk/software/figtree/).

**Table 2:** Genbank Accession number and source of isopod species used for the phylogenetic analysis

Species	Genbank Accession no	Source
*Acanthamunnopsis longicornis* (Hansen, 1895)	EF682265	Osborn (2009)
*Munnopsis abyssalis* Menzies & George, 1972	EF682273	Osborn (2009)
*Paramunnopsis* sp.* 1*	EF682267	Osborn (2009)
*Eurycope glabra* Kensley, 1978	EF682280	Osborn (2009)
*Eurycope complanata* Bonnier, 1896	EF682281	Osborn (2009)
*Dubinectes acutitelson* (Menzies, 1962)	EF682294	Osborn (2009)
*Disconectes antarcticus* (Vanhöffen, 1914)	EF682293	Osborn (2009)
*Tytthocope* sp.* 3*	EF682290	Osborn (2009)
*Munnopsurus* sp.* 1*	EF682289	Osborn (2009)
*Munneurycope murrayi* (Walker, 1903)	EF682275	Osborn (2009)
*Munneurycope* sp.	EF682283	Osborn (2009)
*Paropsurus giganteus* Wolff, 1962	EF682287	Osborn (2009)
*Betamorpha fusiformis* (Barnard, 1920)	EF682291	Osborn (2009)
*Syneurycope heezeni* Menzies, 1962	EF682295	Osborn (2009)
*Ilyarachna antarctica* Vanhöffen, 1914	EF682299	Osborn (2009)
*Notopais cryophila* sp. nov	OL661185	Present study
*Notopais polaris* sp. nov	OL661186	Present study
*Vanhoeffenura* sp.	EF682284	Osborn (2009)
*Acanthocope galatheae* Wolff, 1962	EF682285	Osborn (2009)
*Santia* sp.	AY857831	Lindquist et al. (2005)

## Results

### Taxonomy

**Munnopsidae Lilljeborg, 1864**

**Ilyarachninae Hansen, 1916**

***Notopais*** **Hodgson, 1910**

*Notopais* Hodgson, 1910: 69.— Merrin 2004: 3–4.— Merrin 2007: 131–132. Type species *Notopais spicatus* Hodgson, 1910, by monotypy.

**Diagnosis (updated from Merrin **2004**): **Slender cephalon with frons, leading to an almost horizontal appearance of the maxillipeds in situ in lateral view. Anterior margins of pereonites 1–4 with spines or with robust setae; spines always tipped with robust (pedestal) setae, making them appear more pointed. Horn-like distinctive spine and varying numbers of terminal robust setae on article 1 of antenna 2. Without mandibular palp; large incisor processes, rounded; reduced or absent *lacinia mobilis.* Carpus and propodus of pereopods 5 and 6 flat, expanded, with plumose setae on the margins. Ambulatory pereopods 7 robust, with plumose setation similar to that of pereopods 5 and 6. Biramous uropods (Merrin 2004).

**Description of the genus:** Body robust; frons of cephalon broad, oblong, ventrally flattened, leading to a nearly horizontal appearance of the maxillipeds in lateral view; pereonites 1–4 anterior margins usually with spines, if not, margins with short stiff setae; robust (pedestal) setae on top of every spine; natasome streamlined, not reduced; pleon without anterolateral spines. Antennae positioned closely together; Antenna 2 article 1 distolateral with at least one horn-like spine and varying numbers of terminal robust setae. Mandible with or without spine row, calcareous; *lacinia mobilis* absent or largely reduced; palp absent; large, curved incisor processes, without teeth. Non-enlarged ambulatory pereopod 2, prolonged ischium; pereopods 5 and 6 natatory, carpus paddle-shaped, flat; propodus expanded, flat; dactyli distally narrowing; ischium, carpus and propodus with large plumose setae; ambulatory pereopod 7, robust, long, similarly plumose setation as in pereopod 5 and 6. Pleon vaulted, in dorsal view triangular. Operculum vaulted, bearing a distinctive medial keel. Exopod of pleopod 4 with one or more plumose setae. Uropods biramous (Merrin 2004, 2007).

**Remarks: ***Notopais* can be recognized by its mouthparts, which appear flattened in situ, the anterior marginal spines or robust setae on pereonites 1–4, the long and plumose setation of pereopod 5–7, as well as the horn-like spine on article 1 of antenna 2, which is distinct and unique for this genus, as well as the biramous uropods (Merrin 2004).

*Notopais* and *Echinozone* G. O. Sars, 1897 differ in body shape, being more vaulted in *Echinozone* than in *Notopais*. Besides, *Notopais* can be distinguished from *Echinozone* by the narrower cephalon, the presence of pedestal setae and the exclusive shape of the spine on antenna 2 article 1. This spine resembles a bull´s horn and it is tipped with a robust seta, making it appear more pronounced (Merrin, 2004).

**Composition:** *Notopais beddardi* Merrin, 2004; *Notopais chathamensis* Merrin, 2024; *Notopais echinatus* Merrin & Bruce, 2006; *Notopais euaxos* Merrin & Bruce, 2006; *Notopais likros* Merrin, 2024; *Notopais magnifica* (Vanhöffen, 1914); *Notopais minya* Merrin, 2004; *Notopais quadrispinosa* (Beddard, 1886); *Notopais spinosa* (Hodgson, 1902); *Notopais spicatus* Hodgson, 1910; *Notopais zealandica* Merrin, 2004; and the two new species described in the present work.

The genus *Notopais* is restricted to the Southern Hemisphere, ranging from the SO to the south-eastern continental slope of Australia. It has been found at depths between 36 and 1640 m (Merrin 2007).

***Notopais cryophila*** **sp. nov. Di Franco, Krüger & Brandt, 2026**

**ZooBank LSID: urn:lsid:zoobank.org:act:8606F8E8-42DA-467D-B362-96069AF429CF**

**Fig. 4:**
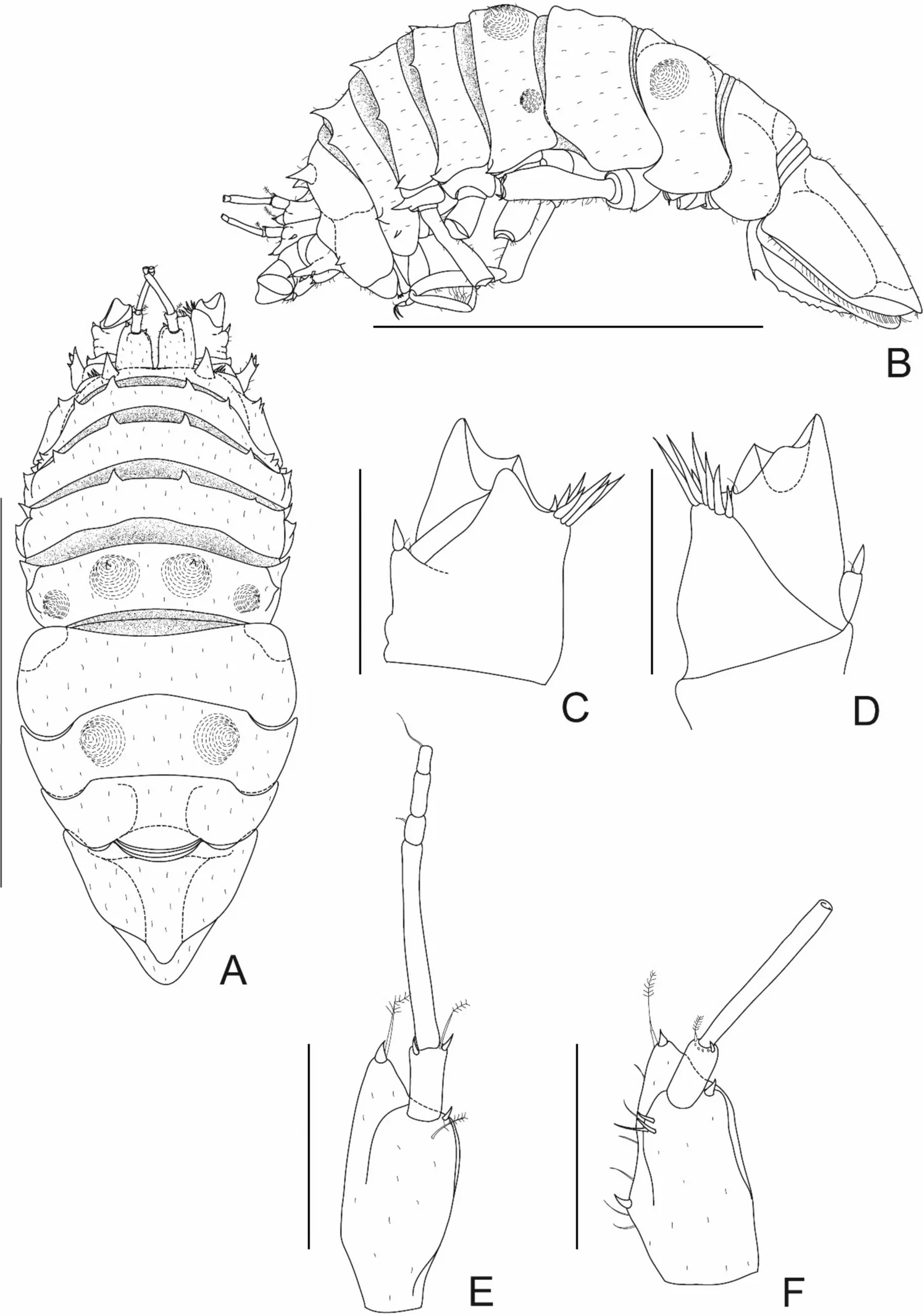
*Notopais cryophila* sp. nov. holotype (SMF-58528), adult female, 4.75 mm: **A** dorsal view; **B** lateral view; paratype (SMF-58530), adult female: **C** dorsal view of right antenna 2 basis; **D** ventral view of right antenna 2 basis; **E** dorsal view of right antenna 1; allotype (SMF-58529), adult male: **F** dorsal view of right antenna 1. Scale bars: **A**, **B** = 3 mm; **C**–**F** = 0.5 mm

**Fig. 5:**
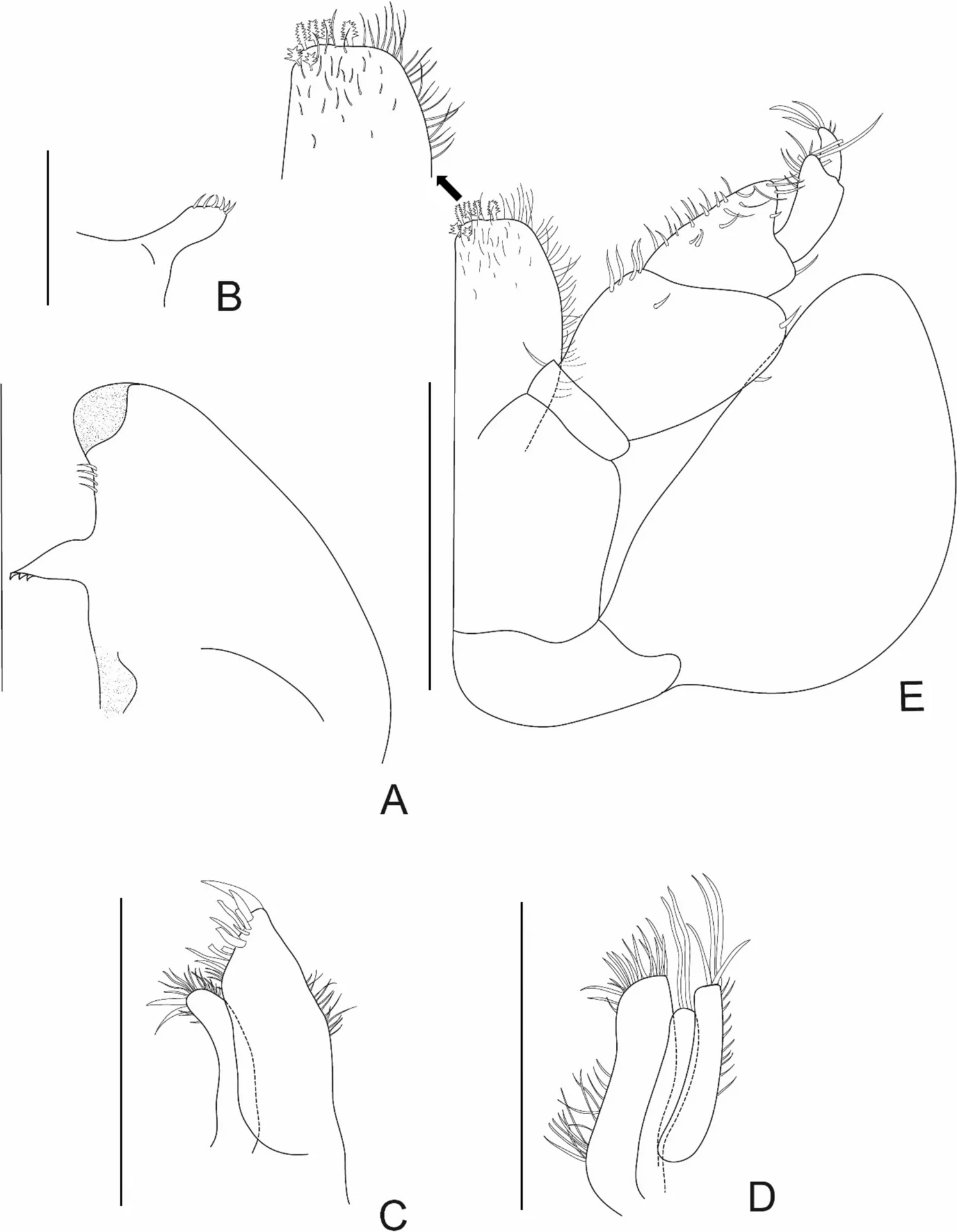
*Notopais cryophila* sp. nov. paratype (SMF-58530), adult female: **A** left mandible; **B** left mandibular molar; **C** left maxilla 1; **D** left maxilla 2; **E** left maxilliped. Scale bars: **A**, **C**–**E** = 0.5 mm; **B** = 0.25 mm

**Fig. 6:**
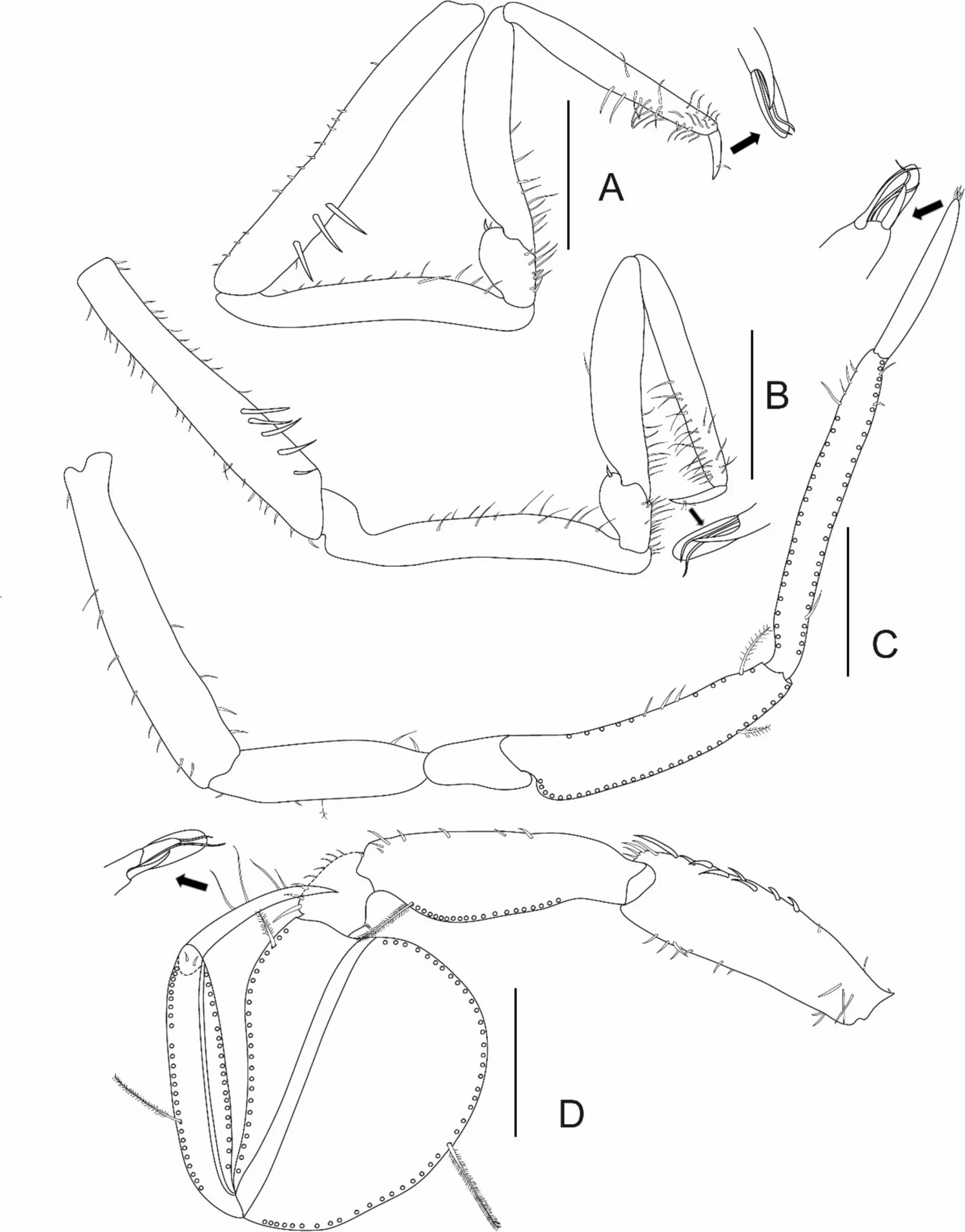
*Notopais cryophila* sp. nov. paratype (SMF-58530), adult female: **A** left pereopod 1; **C** left pereopod 7; **D** right pereopod 6; allotype (SMF-58529), adult male: **B** left pereopod 1. Scale bars: **A**–**D** = 0.5 mm

**Fig. 7:**
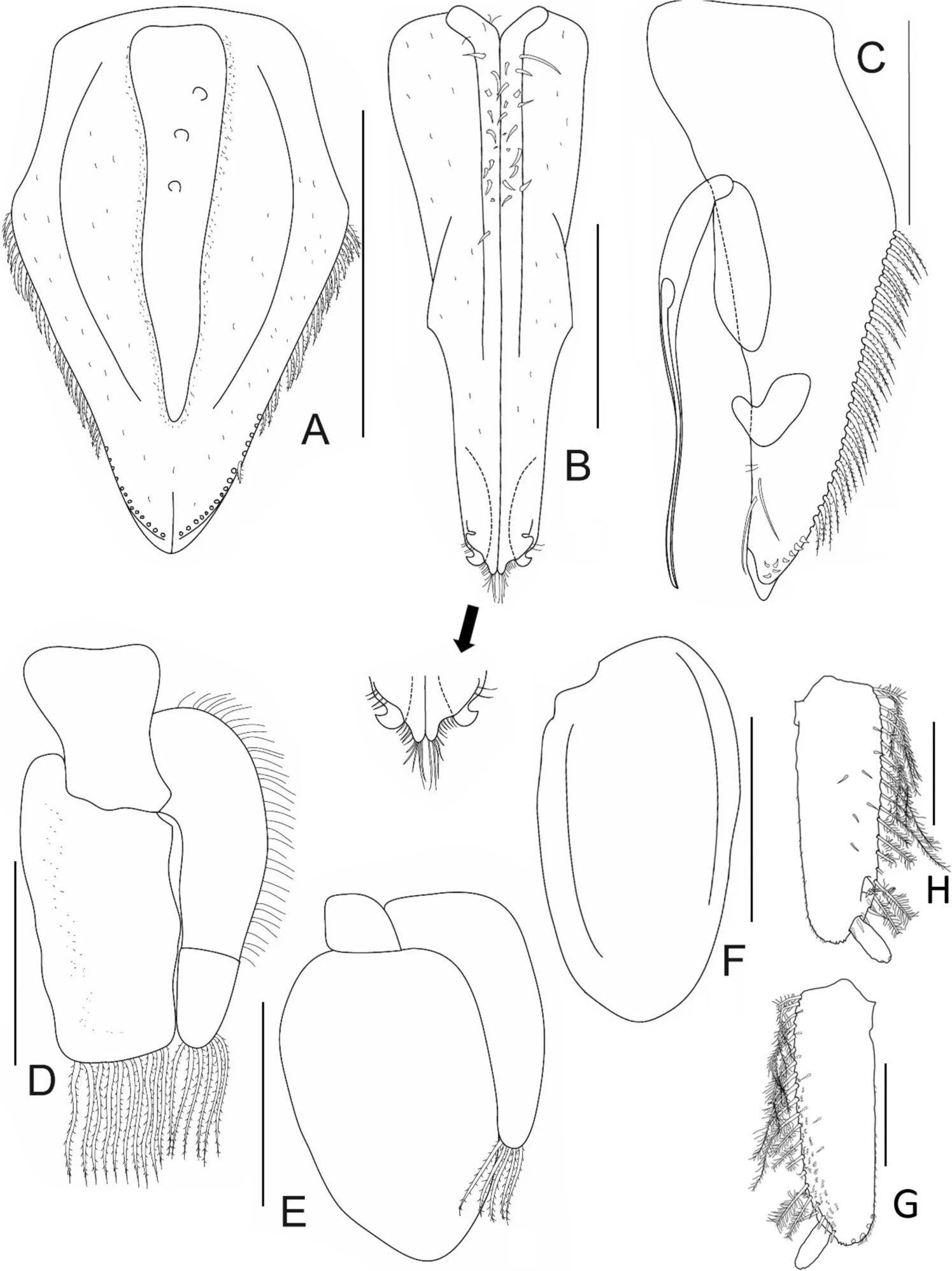
*Notopais cryophila* sp. nov. paratype (SMF-58530), adult female: **A** operculum; **D** left pleopod 3; **E** left pleopod 4; **F** left pleopod 5; allotype (SMF-58529), adult male: **B** pleopods 1; **C** left pleopod 2; **G** posterior view of uropod; **H** anterior view of uropod. Scale bars: **A** = 1 mm; **B**–**F** = 0.5 mm; **G**, **H** = 0.2 mm

**(**Figs. 4, 5, 6, and 7**)**

**Diagnosis of the new species:** Pereonites 1–3 with spines on the anterior margin. Pereonites 4–7 without anterior marginal spines, smooth. Pereonites 1–4 laterally with few small spines. Pereonite 4 dorsally with four tubercles, inner pair with two spines. Pereonite 6 with two tubercles. Cephalon with two large spines.

**Type fixation:** Holotype: adult female SMF-58528, 4.75 mm, designated here.

**Material examined:** Holotype: adult female SMF-58528, 4.75 mm, Filchner Trough, Weddell Sea, Antarctica, RRS *James Clark Ross*, expedition JR275, EBS, Station 109, 75°14.46′S–27°51.72′W, 413–416 m, 4 March 2002. Allotype: adult male SMF-58529, 6.1 mm, Filchner Trough, Weddell Sea, Antarctica, RRS *James Clark Ross*, expedition JR275, EBS, Station 45, 75°45.72′S–30°26.56′W, 428–429 m, 22 February 2002. Paratype: adult female SMF-58530, 7.3 mm, same locality as holotype.

Additional material: 4 specimens, same locality as holotype; 3 specimens, Filchner Trough, Weddell Sea, Antarctica, RRS *James Clark Ross*, expedition JR275, EBS, Station 23; 5 specimens, Filchner Trough, Weddell Sea, Antarctica, RRS *James Clark Ross*, expedition JR275, EBS, Station 45; 3 specimens, Filchner Trough, Weddell Sea, Antarctica, RRS *James Clark Ross*, expedition JR275, EBS, Station 50.

**Type locality:** JR275 station 109, RRS *James Clark Ross*, 413–416 m, 75°14.46′S–27°51.72′W, Filchner Trough, Weddell Sea.

**Distribution:** Filchner Trough, south-eastern Weddell Sea, Antarctica.

**Etymology:** The name *N. cryophila* sp. nov. is related to the ecology of this species, since it was mostly found at stations with cold ice-shelf water, therefore it may have a high affinity with very low temperatures. Cryo is the Latinized form of the Ancient Greek word *kryos* meaning “icy cold” and phila, derived from the Ancient Greek word *phileo* which stands for “having affection for.”

**Description of the female**

**Body (Fig.** 4A, B**).** Body length 4.75 mm. Body 2.3 times as long as pereonite 3, the widest pereonite measured from spine to spine; body highly setose.

**Cephalon (Fig.** 4A–E**).** Lightly setose, with two dorsal spines; anterolateral margins with two small spines; posterolateral margins rounded; ridge encompassing antennae, with no extension.

**Pereonites (Fig.** 4A, B). Pereonites 1 and 2 anterior margins each with four well developed spines; pereonite 3 with two well developed anterior marginal spines and one small spine; pereonite 4 anterior margin smooth with four dorsal tubercles, inner pair of tubercles with two small spines; pereonite 5 anterior margin smooth; pereonite 6 with two dorsal tubercles; pereonite 5–7 anterolateral margins pointing towards cephalon; pereonites 1 and 2 each with pair of widely spaced, small, dorsal spines near lateral margins; pereonites 3 and 4 each with one small, dorsal spine near lateral margin.

**Antenna 1 **(Fig. 4E). **Likely broken at article 6,** comprising 3 peduncular, one small ring-like article and 2 flagellar ones; article 1 distally triangular, 2.3 times as long as wide, distal margin with one robust and one broom seta; article 2 0.3 times as long as article 1, 2.3 times as long as wide, distal margin with two robust seta and one broom seta; article 3 without setae; flagellar article 6 with one distal aesthetasc.

**Antenna 2 (Fig.** 4A–D**).** Article 1 and 2 of antenna 2 broken; article 1–3 more or less triangular, each wider than long; article 1, distolateral angle with three spines; article 2 0.7 times as long as article 1, distolateral angle with one spine; article 3 1.3 times as long as article 1, distolateral angle with one spine and two simple setae, distomedial margin with one short robust seta and seven long robust setae; article 4 1.7 times as long as article 1, distomedial margin with one simple seta.

**Mandible (Fig.** 5A, B**).** Left mandible slightly broader than the right mandible; mandible with four simple setae on mesial margin, spine row absent; molar small, terminated with six short simple setae.

**Maxilla 1 (Fig.** 5C**).** Lateral lobe 2.4 times as wide as medial lobe, lateral margin with 13 fine simple setae, distal margin with four fine simple setae and 11 robust setae; mesial lobe with three fine simple setae on mesial margin, distal margin with two robust setae and 14 fine simple setae.

**Maxilla 2 (Fig.** 5D**).** Lateral lobe with ten fine simple setae, distally with two smaller and two long simple setae; middle lobe 0.7 times as wide as lateral lobe, distally with two long simple setae; mesial lobe 1.6 times as wide as lateral lobe, mesial margin with 13 fine simple setae, terminated with 13 long simple setae and three short simple setae.

**Maxilliped (Fig.** 5E**).** Coxa more or less rectangular, small, 0.4 times as long as wide, 0.2 times as long as basis (including endite); basis elongate, length including endite 2.6 times as long as wide; endite distally with six fan setae (five large, one small), and many fine simple setae; coupling hooks not observed, probably lost/damaged; palp wider proximally, tapers distally, article 1 wide, rectangular, shortest, 0.2 times as long as basal endite, anteromesial margin with one simple seta, article 2 5.1 times as long as, and 1.4 times as wide as article 1, 1.5 times as wide as basal endite; article 3 3.7 times as long as, and 0.9 times as wide as article 1 article 4 almost rectangular, 2.5 times as long as, and 0.4 times as wide as article 1; article 5 small, narrowest of all articles, 1.1 times as long as, and 0.2 times as wide as article 1; epipod elongate, 1.0 times as long as, and 1.9 times as wide as basis length (including endite).

**Pereopods (**Fig. 6A, C, D**)**. Pereopod 1 basis 7.2 times as long as wide, ventral margin with 13 simple setae, dorsal margin with one simple seta; ischium 6.6 times as long as wide, dorsal margin with 11 simple setae; merus 1.4 times as long as wide, ventral margin with ten simple setae, distodorsal margin with one robust and one simple seta; carpus 4.8 times as long as wide, ventral margin with nine long simple setae; propodus 6.8 times as long as wide, ventral margin with nine simple setae, dorsal margin with five simple setae in distal corner; dactylus 5.7 times as long as medial width, distodorsal margin with one simple seta.

**Pereopod 6** basis 3.1 times as long as wide, ventral margin with eight robust setae and seven simple setae, dorsal margin with nine simple setae; ischium 3.4 times as long as wide, ventral margin with seven sub-marginal simple setae, dorsal margin with 20 long plumose setae; merus 1.2 times as long as wide, ventral margin with five simple setae and three robust setae, dorsal margin with two simple setae in distal half; carpus 1.4 times as long as wide, ventral margin with 24 long plumose setae, dorsal margin with 42 long plumose setae; propodus slightly expanded, 4.9 times as long as wide, ventral margin with 20 long plumose setae, dorsal margin with 26 long plumose setae; dactylus 13 times as long as medial width, dorsal margin with four long simple setae.

**Pereopod 7** basis 5.8 times as long as wide, ventral margin with seven simple setae and two sub-marginal simple setae in distal half, dorsal margin with four simple setae; ischium 3.6 times as long as wide, distoventral margin with two simple setae, dorsal margin with two simple setae and one broom seta in proximal half; merus distodorsal margin cup-shaped, 2.2 times as long as wide; carpus 5.4 times as long as wide, ventral margin with three simple setae and at least ten long plumose setae, dorsal margin with 30 long plumose setae; propodus ventral margin with four simple setae in distal half, dorsal margin with three simple setae; dactylus with five simple setae on distal end.

**Pleotelson** **(Fig.** 4A, B**).** 0.7 times as long as proximal width, posterior tip rounded, with scattered simple setae.

**Operculum** **(Fig.** 7A**).** Large, 2.2 times as long as proximal width, distally with medial slit, median keel provided with three robust setae (broken), entire surface with scattered fine simple setae, lateral margins with numerous long plumose setae.

**Pleopod 3** **(Fig.** 7D**).** Exopod narrowing, 1.0 times as long as endopod, with row of fine simple setae and distally with six long plumose setae; endopod 2.7 times as long as wide, with ten long plumose setae.

**Pleopod 4** **(Fig.** 7E**).** Exopod slender, shorter than endopod, with its distal point at 0.6 times length of endopod, with five terminal long plumose setae; endopod oval, 1.5 times as long as wide.

**Pleopod 5** **(Fig.** 7F**).** Oval, 1.9 times as long as wide.

**Description of the male**

**Body.** Body length 6.1 mm, widest at pereonite 3; similar to the female in shape and setation, but narrower than female.

**Cephalon.** Lightly setose, less bulk than in female, with two dorsal spines; anterolateral margins with one small spine; posterolateral margins rounded, similar to the female.

**Pereonites.** Shape similar to the female, but spines are smaller.

**Antenna 1 (Fig.** 4F**).** Broken at article 3, article 1 distal end triangular,

2.0 times as long as wide distal margin with one robust and one broom seta as in female; article 2 0.3 times as long as article 1, 2.0 times as long as wide, distal margin with only one robust seta and one broom seta; article 3 rectangular, longer than article 2.

**Pereopod 1 (Fig.** 6B**).** Basis 6.4 times as long as wide, ventral margin with 21 simple setae, dorsal margin with 11 simple setae; ischium 7.0 times as long as wide, dorsal margin with only eight simple setae (scattered), lateral surface with four simple setae, distoventral margin with three simple setae; merus 1.5 times as long as wide, ventral margin with 11 simple setae (varying in length, scattered), distodorsal margin only with one robust seta; carpus 3.7 times as long as wide, ventral margin with only eight long simple setae in proximal half and one long simple seta on dorsal margin; propodus 7.2 times as long as wide, lateral surface with 17 long simple setae in distoventral half and five long simple setae in distodorsal half; dactylus 6.6 times as long as medial width, distodorsal margin with three simple setae.

**Pleopod 1 (Fig.** 7B**).** 2.8 times as long as proximal width, lateral margins indent 0.2 from distal end, either side of center margin with 27 simple setae (11+16) in proximal half, distally with pair of lobes which go over distolateral horns, with 27 simple setae (14+13).

**Pleopod 2 (Fig.** 7C**).** Sympod 3.6 times as long as wide, lateral margin with 28 long plumose setae (one broken), eight short simple setae in distolateral half; exopod elongate, length 0.1 times as long as sympod; stylet elongate 0.7 times as long as sympod, terminating to a point; sperm duct 0.4 times as long as stylet.

**Uropod (Fig.** 7G, H**).** Protopod 3.2 times as long as wide, distal end rounded; lateral margin with 17 plumose setae and one robust seta. Posterior side (Fig. 7G) of the protopod with a distal row of robust setae near the lateral margin; this area also covered with very small simple setae. Surface with three scattered simple setae; distomesial margin with four visible sockets, but type of setae unknown. Anterior side (Fig. 7H) with seven scattered simple setae. Exopod very small, 0.06 times as long as protopod and 0.3 times as long as the endopod, bearing four simple setae. Endopod 0.2 times as long as the protopod, with one broken simple seta and two visible sockets with missing setae of unknown type.

**Remaining appendages:** Same as in female.

**Remarks: ***Notopais cryophila* sp. nov. is characterized by a set of autapomorphic characters, being the only described Southern Ocean species of *Notopais* lacking dorsal spines on pereonite 4, except for two small spines located on top of the inner pair of the four tubercles. Together with the presence of two tubercles on pereonite 6, these characters distinguish the new species from all other congeners.

*Notopais cryophila* sp. nov. shares some characters with *N. quadrispinosa* (Figs. 2E and 3E), with spines positioned along the edges of the anterior margins of the pereonites, which are less acutely angled than those observed in *N. spicatus* (Figs. 2B and 3B). However, it lacks the spines on pereonite 5 typical of *N. beddardi* Merrin 2004, as well as the characteristic row of short, stout setae present in that species.

***Notopais polaris*** **sp. nov. Di Franco, Krüger & Brandt, 2026**

**ZooBank LSID: urn:lsid:zoobank.org:act:15A8EAB3-B32B-4427-9D56-E20C4DD83247**

**Fig. 8:**
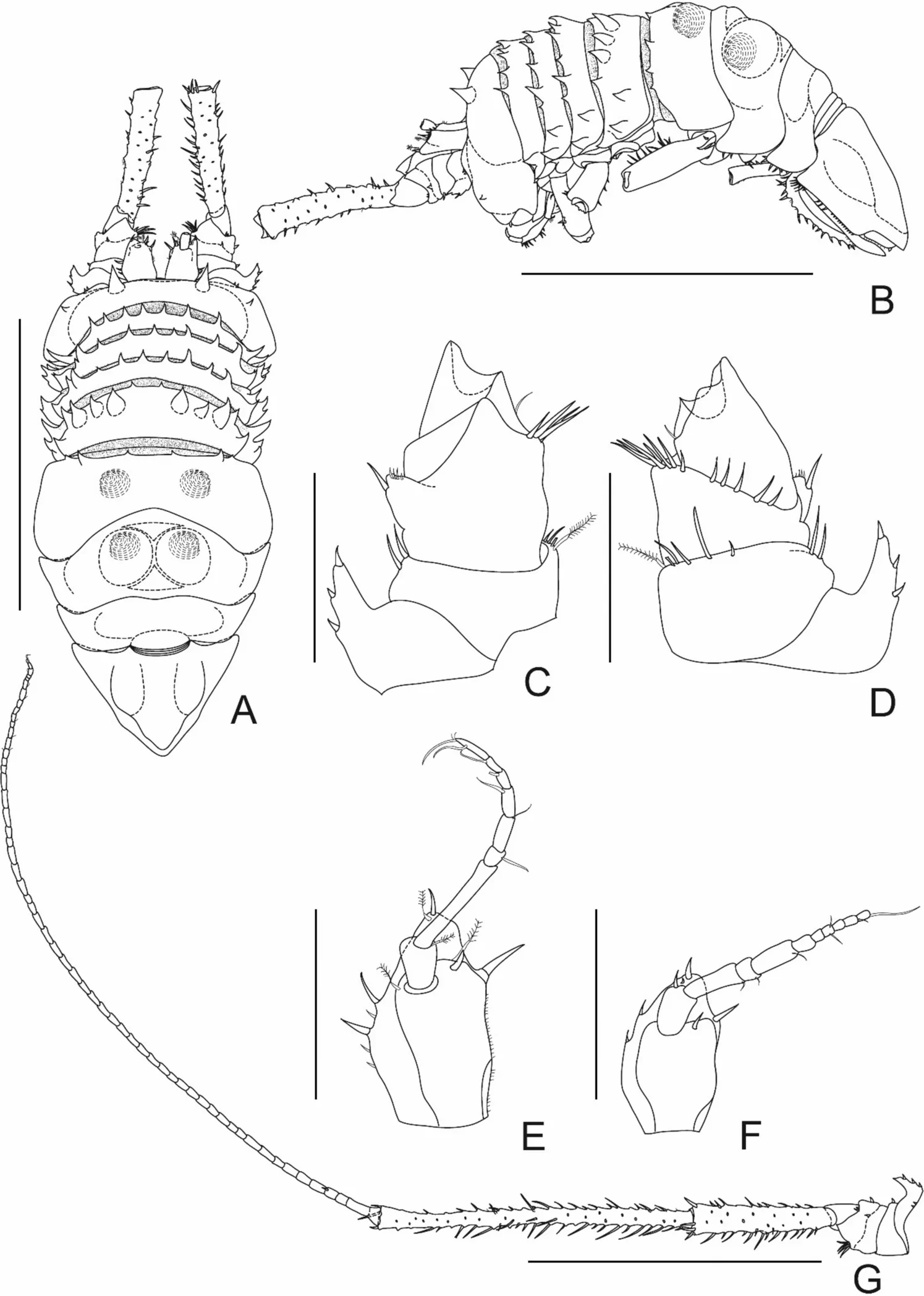
*Notopais polaris* sp. nov. holotype (SMF-58531), adult female, 4.90 mm: **A** dorsal view; **B** lateral view; **G** dorsal view of right antenna 2; paratype (SMF-58533), adult female: **C** right antenna 2, dorsal view; **D** right antenna 2, ventral view; **E** right antenna 1, dorsal view; allotype (SMF-58532), adult male: **F** right antenna 1, dorsal view. Scale bars: **A**, **B**, **G** = 3 mm; **C**–**F** = 0.5 mm

**Fig. 9:**
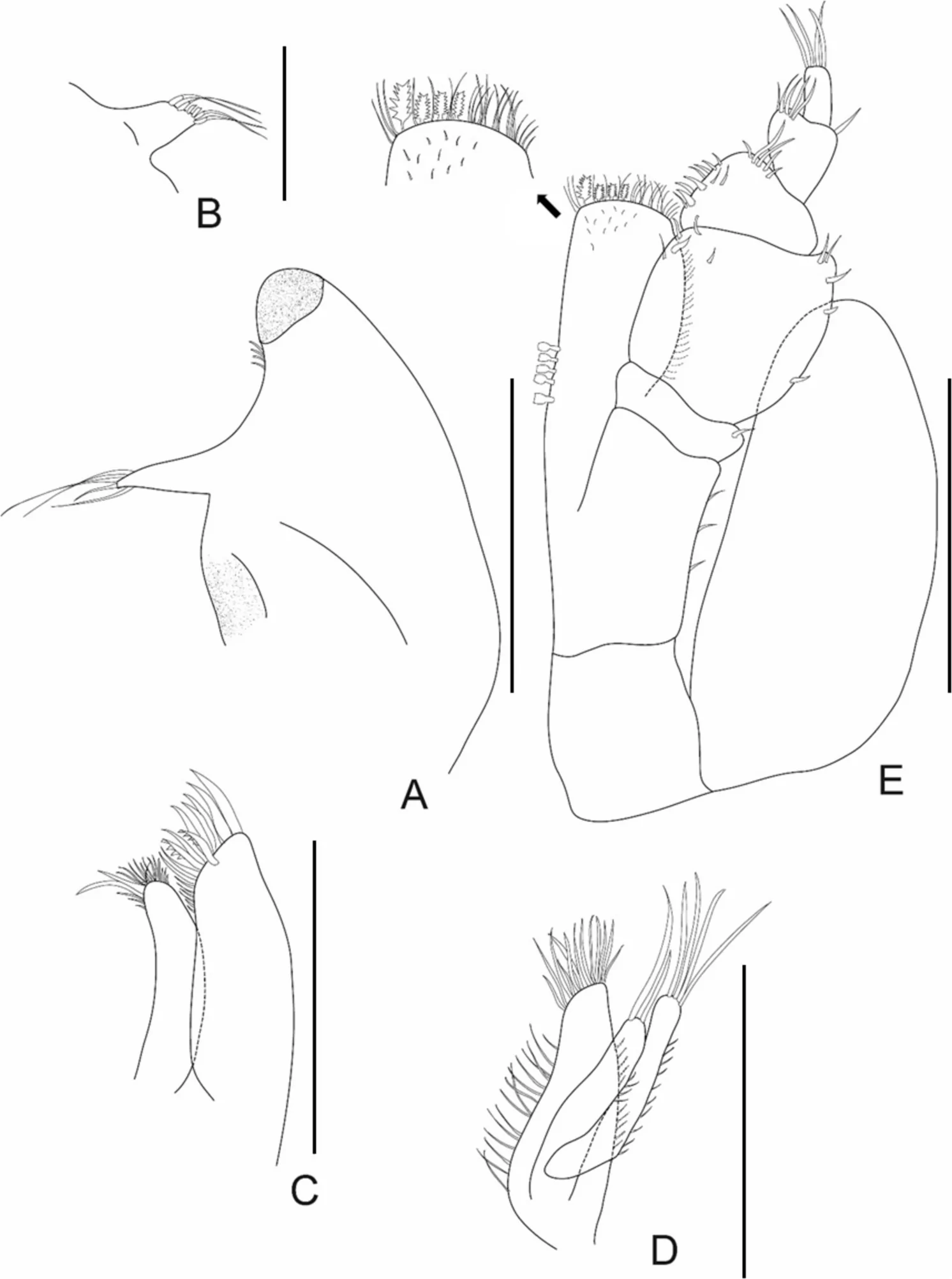
*Notopais polaris* sp. nov. paratype (SMF-58533), adult female: **A** left mandible; **B** left mandibular molar; **C** left maxilla 1; **D** left maxilla 2; **E** left maxilliped. Scale bars: **A**, **C**–**E** = 0.5 mm; **B** = 0.25 mm

**Fig. 10:**
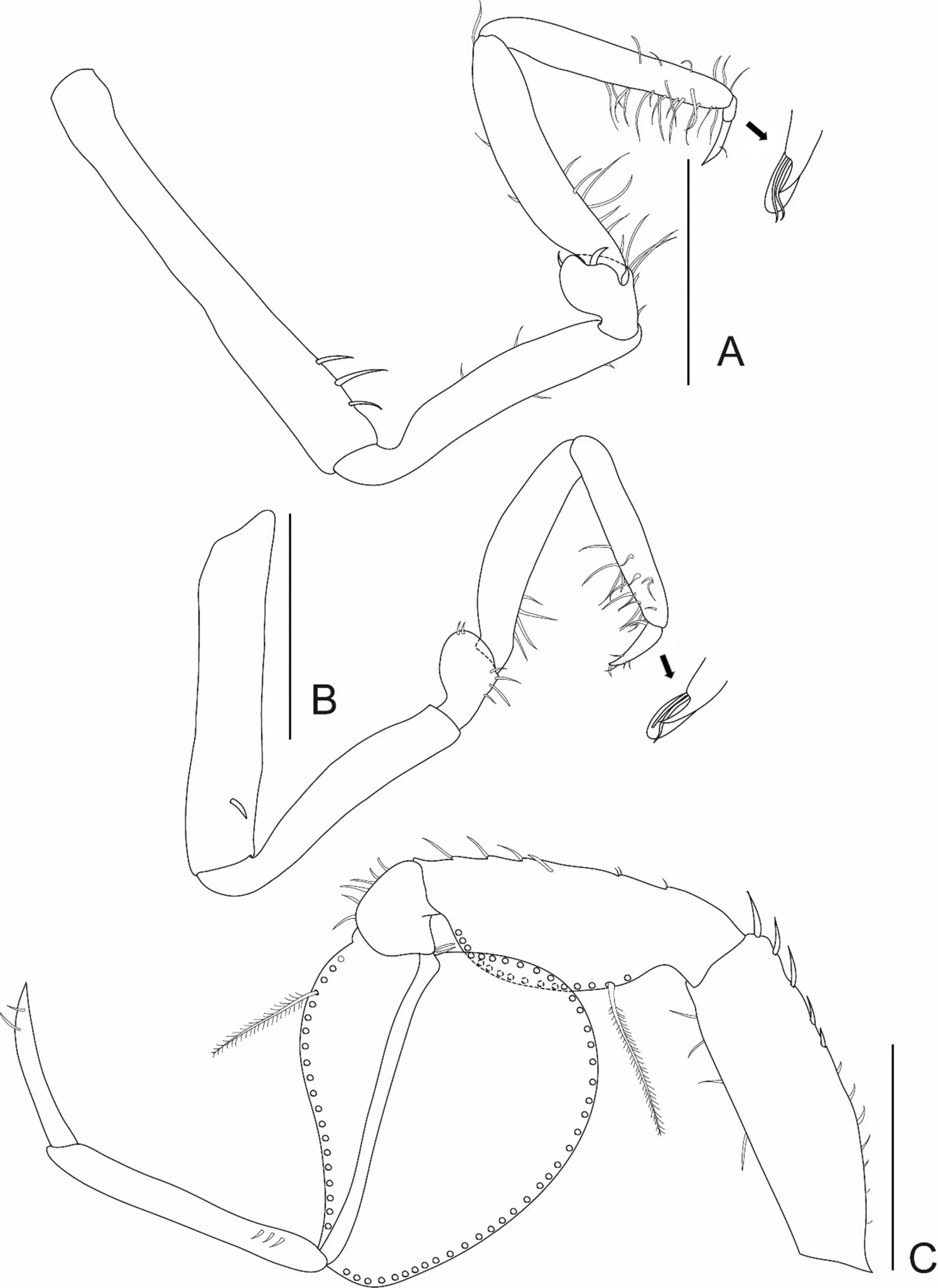
*Notopais polaris* sp. nov. paratype (SMF-58533), adult female: **A** left pereopod 1; **C** right pereopod 6; allotype (SMF-58532), adult male: **B** left pereopod 1. Scale bars: **A**–**C** = 0.5 mm

**Fig. 11:**
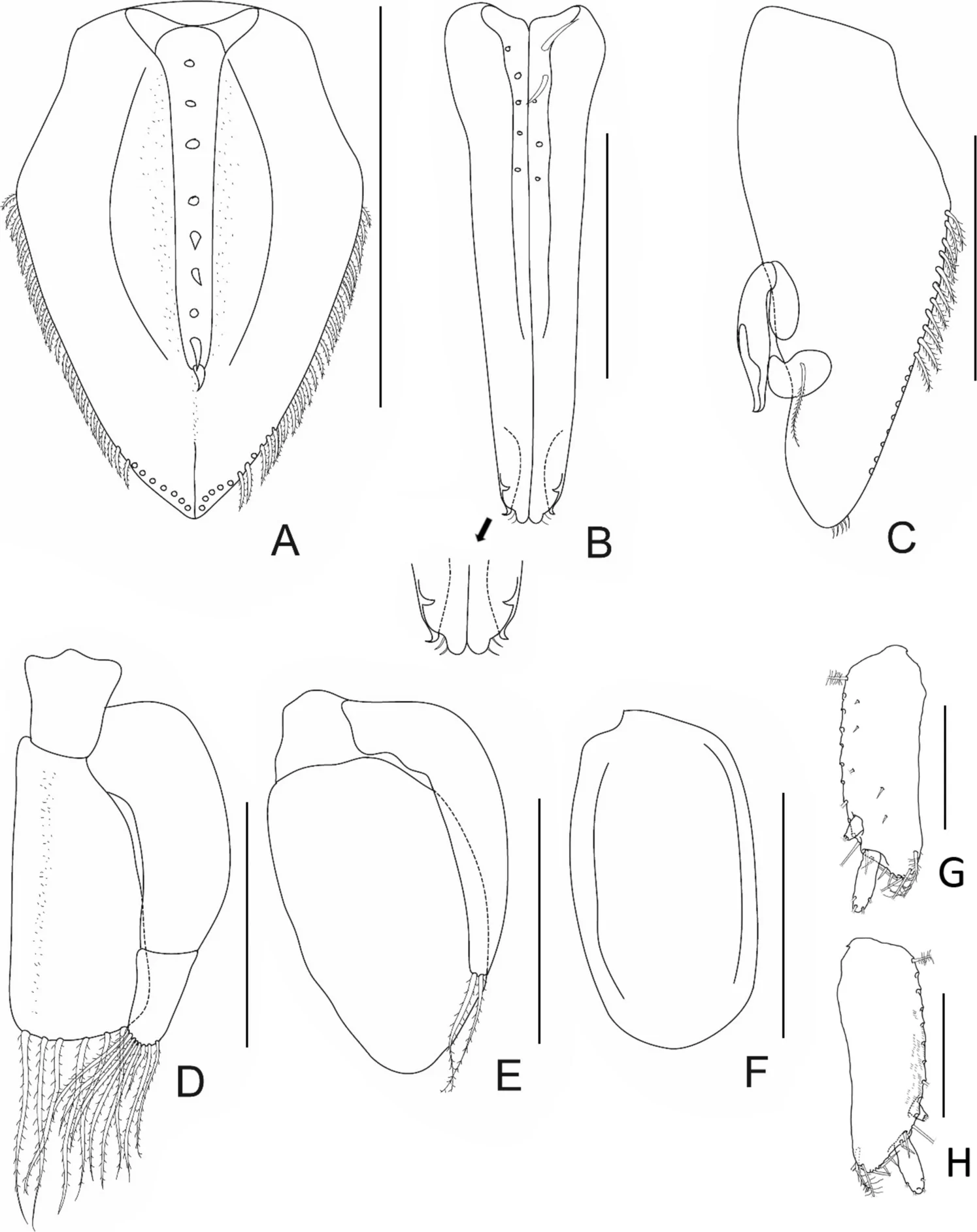
*Notopais polaris* sp. nov. paratype (SMF-58533), adult female: **A** operculum; **D** left pleopod 3; **E** left pleopod 4; **F** left pleopod 5; allotype (SMF-58532), adult male: **B** pleopods 1; **C** left pleopod 2; **G** anterior view of uropod; **H** posterior view of uropod. Scale bars: **A** = 1 mm; **B**–**F** = 0.5 mm; **G**, **H** = 0.2 mm

**(Figs.** 8, 9, 10, and 11**)**

**Diagnosis of the new species:** Pereonites 1–5 with spines on the anterior margin. Pereonite 4 with large dorsal spines. Pereonites 6 and 7 without spines. Pereonites 1–4 each with one pair of small spines laterally. Pereonite 5 and 6 with two tubercles dorsally. Cephalon with two large spines and two small sub-marginal spines in anterolateral half.

**Type fixation:** Holotype: adult female SMF-58531, 4.9 mm, designated here.

**Material examined:** Holotype: adult female SMF-58531, 4.9 mm, South Orkney Islands, Antarctica, RRS *James Clark Ross*, expedition JR15005, EBS, Station 34, 62°09.61′S–44°58.92′W, 524–561 m, 6 March 2016. Allotype: adult male SMF-58532, 4 mm, same locality as holotype. Paratype: adult female SMF-58533, 4.7 mm, same locality as holotype.

Additional material: 7 specimens, same locality as holotype; 1 specimen, South Orkney Islands, Antarctica, RRS *James Clark Ross*, expedition JR15005, EBS, Station 103; 4 specimens, South Orkney Islands, Antarctica, RRS *James Clark Ross*, expedition JR15005, EBS, Station 133.

**Type locality:** JR15005 station 34, RRS *James Clark Ross*, 524–561 m, 62°09.61′S–44°58.92′W, South Orkney Islands, Antarctica.

**Distribution:** South Orkney Islands, Antarctica.

**Etymology:** *Notopais polaris* sp. nov. has a large number of spines, a shape resembling a star. Derived from the Latin words *stella polaris*, meaning “pole star,” *N. polaris* sp. nov. is named after its geographical distribution.

**Description of the female**

**Body (Fig.** 8A, B**).** Body length 4.90 mm; in total 2.1 times as long as the greatest width (from spine to spine) of widest pereonite; widest at pereonite 4.

**Cephalon (Fig.** 8A–E, G**).** With two dorsal spines and one small sub-marginal spine on each side in antero-marginal half; posterolateral margins rounded; ridge encompassing antennae, with no extension.

**Pereonites (Fig.** 8A, B**).** Pereonites 1–3 anterior margins with continuous row of spines; pereonite 4 with four small anterior marginal spines and six well developed anteriorly directed dorsal spines; pereonites 1–4 each with pair of widely spaced, small, dorsal spines near lateral margins; pereonite 5 with four spines on anterior margin; pereonite 5 and 6 dorsally with two tubercles; pereonite 5–7 anterolateral margins pointing towards cephalon.

**Antenna 1 (Fig.** 8E**).** Antenna 1 of 9 articles (number of articles, that were actually visible); article 1 1.7 times as long as wide, distal margin rectangular, with one robust and one broom seta; article 2 0.3 times as long as article 1, 1.6 times as long as wide, distal margin with one broom seta; article 4–8 each with one simple seta; article 9 distally with two fine simple setae and two aesthetascs.

**Antenna 2 (Fig.** 8A–D, G**).** Article 1 and 2 more or less triangular, both wider than long; article 3 pentagonal, longer than wide; article 1 lateral margin with three spines; article 2 0.6 times as long as article 1, distomedial margin with one broom seta; article 3 1.2 times as long as article 1; article 4 1.0 times as long as article 1; article 5 3.6 times as long as article 1; article 6 8.0 times as long as article 1; flagellum of 56 articles, distalmost article with two aesthetascs.

**Mandible (Fig.** 9A, B**).** Left mandible slightly broader than the right mandible; mandible with four simple setae on mesial margin, spine row absent; molar small, terminated with six long simple setae.

**Maxilla 1 (Fig.** 9C**).** Lateral lobe 2.0 times as wide as medial lobe, distal margin with six fine simple setae, two robust dentate setae and eight robust setae; mesial lobe with three fine simple setae on mesial margin, distal margin with two robust setae and 13 fine simple setae.

**Maxilla 2 (Fig.** 9D**).** Lateral lobe with ten fine simple setae on lateral margin, distally with four long simple setae (same length); middle lobe 1.2 times as wide as lateral lobe, lateral margin with four simple setae, distally with two long simple setae (one shorter than the other); mesial lobe 3.0 times as wide as lateral lobe, mesial margin with 15 long, fine simple setae, lateral margin with ten short, fine simple setae, terminating with ten long simple setae and three short simple setae.

**Maxilliped (Fig.** 9E**).** Coxa rectangular, 1.2 times as long as wide, 0.4 times as long as basis (including endite); basis elongate, length including endite 3.1 times as long as wide, setae; endite with five coupling hooks on mesial margin, distally with four fan setae and many fine simple setae; palp wider proximally, tapers distally, article 1 wide, rectangular, shortest, 0.2 times as long as basal endite; article 2 5.3 times as long as, and 1.3 times as wide as article 1, 1.8 times as wide as basal endite; article 3 2.0 times as long as, and 0.9 times as wide as article 1; article 4 heart shaped, 2.2 times as long as, and 0.5 times as wide as article 1; article 5 rectangular, narrowest of all articles, 1.5 times as long as, and 0.2 times as wide as article 1; epipod elongate, 1.1 times as long as, and 1.5 times as wide as basis length (including endite).

**Pereopods (Fig.** 10A, C**).** Pereopod 1 basis 8.5 times as long as wide, dorsal margin with three distal robust setae; ischium 6.5 times as long as wide, dorsal margin with two simple setae, ventral margin also with two simple setae; merus 1.4 times as long as wide, ventral margin with five simple setae, distodorsal margin with one simple seta, and distal margin with one robust seta; carpus 5.2 times as long as wide, ventral margin with seven long simple setae, two simple setae on dorsal margin, and one long simple seta on distodorsal margin; propodus 7.5 times as long as wide, ventral margin with 13 simple setae, dorsal margin with four simple setae in distal corner; dactylus 5.3 times as long as proximal width, distodorsal margin with one simple seta.

**Pereopod 6** basis 2.8 times as long as wide, ventral margin with five robust setae, five simple setae and two fine simple setae, dorsal margin with four long simple setae; ischium 2.6 times as long as wide, ventral margin with four simple setae in distal half and three fine simple setae in proximal half, dorsal margin with 16 long plumose setae; merus 0.9 times as long as wide, ventral margin with six simple setae; carpus 1.1 times as long as wide, ventral margin with 20 long plumose setae; propodus elongated, 8.1 times as long as wide; dactylus six times as long as proximal width, lateral surface with two simple setae in distodorsal corner.

**Pleotelson (Fig.** 8A, B**).** 0.6 times as long as proximal width, posterior tip rounded, lateral margins with ventrally facing robust setae.

**Operculum (Fig.** 11A**).** Large, 2.2 times as long as proximal width, distally with medial slit, median keel provided with nine robust setae (five broken), lateral margins with numerous long plumose setae.

**Pleopod 3 (Fig.** 11D**).** Exopod narrowing, 1.2 times as long as endopod, distally with eight long plumose setae (varying in length); endopod 2.2 times as long as wide, with six long plumose setae.

**Pleopod 4 (Fig.** 11E**).** Exopod slender, shorter than endopod, with two terminal long plumose setae; endopod oval, 1.7 times as long as wide.

**Pleopod 5 (Fig.** 11F**).** Oval, 1.8 times as long as wide.

**Description of the male**

Body. Body length 4.0 mm, widest at pereonite 3; similar to the female in shape, but smaller and narrower than in female.

**Cephalon.** More robust in female, with two dorsal spines; posterolateral margins rounded, similar to female.

**Pereonites.** Shape similar to the female, but spines and tubercles are smaller.

**Antenna 1 (Fig.** 8F**).** Antenna 1 of 11 articles (number of articles that were actually visible); article 1 distal end more rounded than rectangular, 1.7 times as long as wide, distal margin with one robust and one small simple seta instead of one broom seta as in female; article 2 0.3 times as long as article 1, 1.4 times as long as wide, distal margin with one robust seta instead of one broom seta; remaining articles rectangular, varying in length; last article with one aesthetasc on distal margin.

**Pereopod 1 (Fig.** 10B**).** Basis 5.2 times as long as wide, dorsal margin with only one distal robust seta; ischium 6.1 times as long as wide, dorsal margin without setae; merus 1.4 times as long as wide, ventral margin only with four simple setae, distodorsal margin with two simple setae, distal margin without robust setae; carpus 5.3 times as long as wide, ventral margin with only four long simple setae in proximal half, no simple setae on dorsal margin; propodus 5.6 times as long as wide, ventral margin with only 11 simple setae, dorsal margin without simple setae; dactylus 3.5 times as long as proximal width, distodorsal margin with four simple setae, distoventral margin with one simple seta and proximal end of ventral margin with one simple seta.

**Pleopod 1 (Fig.** 11B**).** 3.7 times as long as proximal width, either side of center margin with ten simple setae (5+5) in proximal half, distally with pair of lobes which go over distolateral horns, with four simple setae (2+2).

**Pleopod 2 (Fig.** 11C**).** Sympod 2.8 times as long as wide, lateral margin with 20 long plumose setae (nine broken), and distolateral margin with five fine simple setae; exopod elongate, length 0.1 times as long as sympod, with one plumose seta; stylet 0.3 times as long as sympod, terminating to a point; sperm duct 0.5 times as long as stylet.

**Uropod (Fig.** 11G, H**).** Protopod 2.8 times as long as wide, distal end rounded; lateral margin with one plumose seta and two broken setae (probably plumose), eight sockets visible but type of setae unknown (probably plumose setae), one robust seta. Posterior side (Fig. 11H) of the protopod covered with very little simple setae near the lateral margin; distomesial margin with four plumose setae and two simple setae. Anterior side (Fig. 11G) of the protopod with 5 scattered simple setae. Exopod small, 0.1 times as long as protopod and 0.4 times as long as endopod, with two broken setae. Endopod 0.2 times as long as protopod with four broken setae.

**Remaining appendages:** Same as in female.

**Remarks:** *Notopais polaris* sp. nov. shares some traits with *N. magnifica* (Fig. 2A, Fig. 3A), as it also has continuous rows of spines on the anterior margins of pereonites 1–3. Besides, *N. magnifica* shows anterior marginal spines and dorsal spines on pereonite 4, although these are only two instead of six. *N. quadrispinosa* (Fig. 2E, Fig. 3E) and *N. spinosa* (Fig. 2D, Fig. 3D) also show sub-marginal spines; however, both have only four of them. Moreover, *N. quadrispinosa* has no anterior marginal spines, while *N. spinosa* shows sub-marginal spines only on the posterior margin. These spines in *N. polaris* sp. nov. are situated more medially and much larger. Although *N. polaris* sp. nov. bears four anterior marginal spines on pereonite 5 like *N. spicatus*, *N. spinosa*, and *N. quadrispinosa* (Fig. 2, Fig. 3), the presence of two tubercles on its pereonite 5 is a unique feature. Besides, it presents two additional tubercles on pereonite 6 as solely seen in *N. cryophila* sp. nov., which makes them the only described species of *Notopais* from the Southern Ocean with this feature.

### Genetic results

#### Whole genome sequencing and assembly

Illumina sequencing yielded 573,110,218 reads for *Notopais cryophila* sp. nov. and 624,723,258 reads for *Notopais polaris* sp. nov.. K-mer analysis estimated the genome size of *Notopais cryophila* sp. nov. to 1.44 Gb and 2.59 Gb for *Notopais polaris* sp. nov. The genomic heterozygosity in *Notopais cryophila* sp. nov. was 1.52%, while in *Notopais polaris* sp. nov. it was 0.70%.

**Fig. 12:**
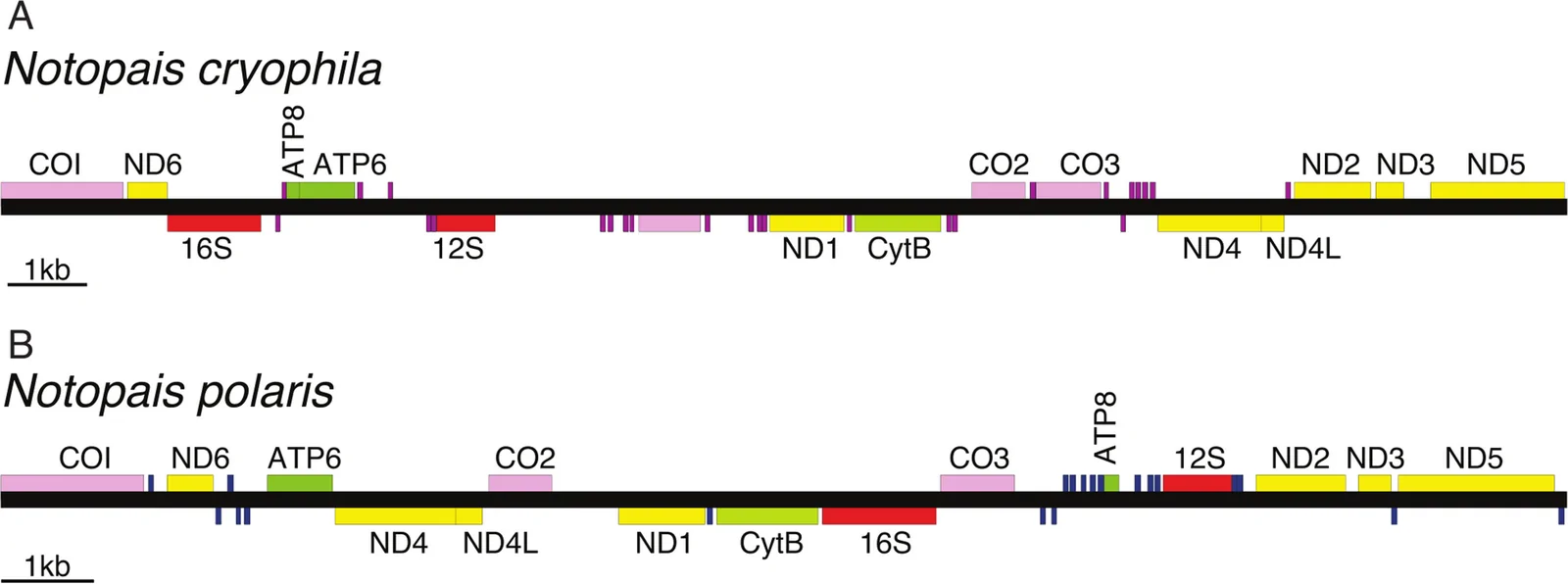
Mitochondrial genome gene arrangement of *Notopais polaris* sp. nov. and *Notopais cryophila* sp. nov.. The mitochondrial genomes have been modified to start at COI. The protein coding genes and ribosomal genes are shown at their location. tRNAs are shown as short colored vertical lines without names as these are uncurated. Genes below the black line are encoded on the opposite strand. The scale bar indicates 1000 bp

The mitochondrial genomes were assembled into one linear sequence each as neither MitoZ nor NOVOPlasty were able to circularize them (21,955 bp *Notopais cryophila* sp. nov.; 19,878 bp *Notopais polaris* sp. nov.). All expected 13 protein coding genes, 2 rRNAs could be annotated on each of the mitochondrial genome sequences. For *N. cryophila* sp. nov., all 20 different expected tRNAs could be annotated of which 8 were found more than once (37 in total). In *N. polaris* sp. nov.’s mitochondrial genome, one of the 20 expected tRNAs (Isoleucine) was not found and 4 tRNAs are annotated at least twice (27 in total). The gene order and arrangement of the mitochondrial genomes are shown in Fig. 12.

The final nuclear *Notopais cryophila* sp. nov. genome assembly contained 544,079 contigs with a total length of 535,477,874 bp. The genome size was estimated to 1.12 Gb and heterozygosity to 1.53% based on k-mers. The back-mapping rate was as high as 98.54% and the genome sequence coverage of the holotype specimen was uniformly distributed at ca. 43X. The BUSCO search resulted in 43.3% present BUSCO loci (C:15.5%[S:15.4%,D:0.1%],F:27.8%,M:56.7%,n:1013).

The final nuclear *Notopais polaris* sp. nov. genome assembly contained 448,606 contigs with a total length of 609,453,175 bp. The genome size was estimated to 2.08 Gb and heterozygosity to 0.713% based on k-mers. The back-mapping rate was as high as 98.59% and the genome sequence coverage of the holotype specimen was uniformly distributed at ca. 25X. The BUSCO search resulted in 58.9% present BUSCO loci (C:21.7%[S:21.7%,D:0.0%],F:37.2%,M:41.1%,n:1013).

Raw reads and the draft nuclear genome assemblies can be found within the BioProject PRJNA780396. The Genbank accession numbers of the mitochondrial genomes are OL661185 for *N. cryophila* sp. nov. and OL661186 for *N. polaris* sp. nov.

### Phylogenetic results

**Fig. 13:**
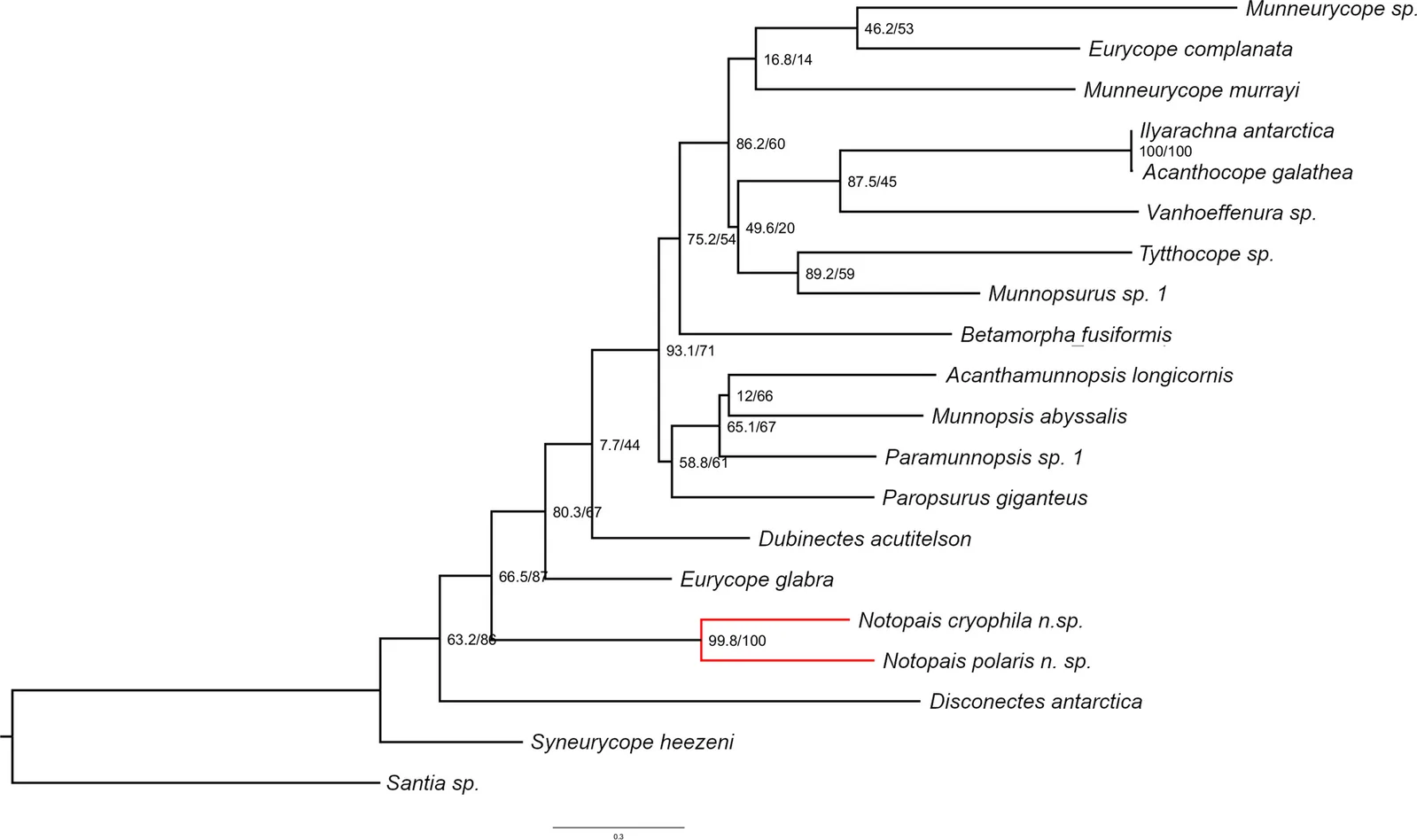
Phylogenetic tree based on COI sequences of 19 species of munnopsid isopods and one outgroup (*Santia* sp.). The two new species of *Notopais* branch are highlighted in red. Node support values are expressed as SH-aLRT support (%)/ultrafast bootstrap support (%)

The CO1 alignment of 20 species of Isopoda (Table 2) was 643 nt. ML analyses reconstructed a phylogeny where *Notopais cryophila* sp. nov. and *Notopais polaris* sp. nov. are sister species with 99.8/100% support (Fig. 13). The internal nodes in the phylogeny received very low support values between 7.7 and 99.8% likely reflecting the deep evolutionary relationship between the species. Pairwise distance estimation using the K2P model calculated that the two new species of *Notopais* have 23.8% sequence distance. Thus, the phylogenetic analysis supports the morphological distinction between the two new *Notopais* species.

## Discussion

The recognition of the two new species is primarily based on distinct morphological characters, including unique combinations of dorsal spines, tubercles, and setation patterns, as detailed in the Diagnosis and Remarks sections. These characters clearly separate the new species from all other known species of *Notopais* occurring in the Southern Ocean. In addition to the Southern Ocean congeners discussed above, the new species can also be distinguished from the remaining species of *Notopais* described from temperate regions off Australia and New Zealand by their unique combination of dorsal spine and tubercle patterns, as well as differences in pereonite armature and setation (Merrin 2004, 2007, 2024; Merrin and Bruce 2006). Previously described Southern Ocean species of *Notopais* exhibit wide, often circum-Antarctic distributions, as illustrated in Fig. 1. In contrast, the two new species described here are currently only known from their respective type localities in the Weddell Sea and the South Orkney Islands.

Whether this reflects true endemism or limited sampling in deep-sea Antarctic habitats remains to be assessed by future studies. Integrative taxonomy analysis provides evidence for two new *Notopais* species from the Southern Ocean based on morphological differences as well as molecular phylogenetics. The two species were subjected to a thorough morphological characterization as well as whole genome sequencing. The obtained sequence data is the first whole genome draft assemblies from the deep-sea Munnopsidae as well as the first mitochondrial sequences from the genus *Notopais*.

Several lines of evidence provide significant support that the two new *Notopais* species should be considered to be separate and are distantly related.

The reported threshold values to delineate intraspecific divergence for isopods are usually below 5%, while 5–10% indicate closely related species, and > 18% should be considered distantly related species (Brix et al. 2011). The two new *Notopais* species had a pairwise distance of 23% based on the mitochondrial CO1 gene. The distinction between the two species was also confirmed by their different genome size of 1.44 Gb and 2.59 Gb as well as large differences in genomic heterozygosity (1.52%/0.70%). This indicates that the two *Notopais* species are significantly distinct on the molecular level and that the observed morphological differences are not due to phenotypic adaptation.

So far, no mitochondrial sequences have been published from the genus *Notopais*. Genetic analyses were previously performed on this genus in (Raupach et al. 2004, 2007, 2009); however, no COI sequences were analyzed. We tried to obtain COI sequences from the two new *Notopais* species using protocol, universal primers, and other COI primers as described in Riehl et al. (2014) without success. DNA barcoding and the use of the COI gene is a successful and widely used tool for the identification of many species in association with classical taxonomy (DeSalle and Goldstein 2019). But it has become evident that the identification of species based on the COI can be problematic; the evolutionary rate of COI varies highly between taxa and for this reason this gene marker has failed for a wide range of marine invertebrates (Rach et al. 2017). Frequently, the universal primers designed for the COI region by Folmer et al. (1994) are replaced with primers that are specific for a certain taxon (Rach et al. 2017). In genetic studies of isopods, universal primers are very often used in association with other COI primers (e.g., Brix et al. 2011, 2014; Riehl et al. 2014; Kaiser et al. 2018; Bober et al. 2019). It has been shown that in the Munnopsidae family the universal primers for COI can have a rate of success of about 50%, lower than that of the mitochondrial 16 S rRNA gene (Riehl et al. 2014).

The use of next-generation sequencing techniques can generate both complete genome and mitochondrial genome sequences. Such information can solve the issue of finding working primers for certain taxa and provides additional genetic information compared to those obtained from traditional mitochondrial barcoding markers such as COI or 16S. Due to this limitation, Köhler et al. (2021) proposed to add genome data for the genetic characterization of holotype specimens, suggesting to use a low-coverage short read–based genome sequence given that the costs for this analyses have significantly decreased (Köhler et al. 2021; Letsch et al. 2025). Recent studies on the complete mitochondrial genomes revealed to be very promising and useful for the systematic and phylogenetic reconstruction of marine isopods (Kilpert and Podsiadlowski 2006; Shen et al. 2017; Yu et al. 2018). In line with the aforementioned authors, we suggest that whenever possible, the use of a complete mitochondrial genome, or a draft genome assembly, in association with the type material description should be considered for isopods. However, it is not always possible to obtain large amounts of DNA from tissue samples. For deep-sea isopods, it is difficult to obtain fresh material due to their remote habitat and issues relating to DNA extraction and amplification were frequently reported, possibly due to active nucleases that quickly digest the DNA (Raupach et al. 2004; Raupach and Wägele 2006; Brix et al. 2011; Riehl et al. 2014). Although recent developments in next-generation technologies allow using a small amount of DNA, often it is necessary to destroy the whole specimen of small animals. However, given the taxonomic importance of holotype specimens, we suggest using paratype specimens whenever it is not possible to obtain sufficient amounts of DNA and to prevent the destruction of holotype material (Letsch et al. 2025).

Our results provide the first nuclear and mitochondrial draft assemblies of isopods from the Southern Ocean. This represents the first step for a more comprehensive genetic characterization of species that will facilitate researchers in obtaining systematic and phylogenetic information on Southern Ocean isopods.

## Supplementary Information

Below is the link to the electronic supplementary material.

(DOCX 14.6 KB)

## Declarations

### Data availability

Gbif data are directly available at the following link: https://doi.org/10.15468/dl.smde95. OBIS data directly available at the following link: https://obis-datasets.ams3.digitaloceanspaces.com/downloads/417708e8-98bc-4eff-949c-c8315f4d7b00.zip. All sequence data is available within the BioProject PRJNA780396, this includes both reads and as well as the draft nuclear genome assemblies. The Genbank accession numbers for the mitochondrial genomes are OL661185 and OL661186.
