# Supplementary material for: Integrative taxonomy and whole genome sequencing of two new isopod species of the genus Notopais Hodgson, 1910 (Crustacea, Malacostraca) from the Southern Ocean
Source: Mar Biodivers. 2026 Nov 12;56(6):122. doi: 10.1007/s12526-026-01727-0 (PMC13759451; doi:10.1007/s12526-026-01727-0)
Supplement: Supplementary file 1 [file 12526_2026_1727_MOESM1_ESM.docx]

| **Specimens** | **Collection-No.** | **Sex** | **Life Stage** | **Length** | **Treatment** |
| --- | --- | --- | --- | --- | --- |
| *N. cryophila* sp. nov. (holotype) | SMF-58528 | female | adult | 4.75 mm | habitus drawings of dorsal and lateral view |
| *N. cryophila* sp. nov. (allotype) | SMF-58529 | male | adult | 6.10 mm | dissected for appendage drawings (antenna 1, pereopod 1, pleopod 1, 2, uropod) |
| *N. cryophila* sp. nov. (paratype) | SMF-58530 | female | adult | 7.30 mm | dissected for appendage drawings (antenna 1, 2, maxilliped, maxilla 1, 2, mandible, pereopod 1, 6, 7, pleopod 3-5, operculum) |
| *N. polaris* sp. nov. (holotype) | SMF-58531 | female | adult | 4.90 mm | habitus drawings of dorsal and lateral view, antenna 2 |
| *N. polaris* sp. nov. (allotype) | SMF-58532 | male | adult | 4.00 mm | dissected for appendage drawings (antenna 1, pereopod 1, pleopod 1, 2, uropod) |
| *N. polaris* sp. nov. (paratype) | SMF-58533 | female | adult | 4.70 mm | dissected for appendage drawings (antenna 1, 2, maxilliped, maxilla 1, 2, mandible, pereopod 1, 6, pleopod 3-5, operculum) |
| *N. magnifica* (Vanhöffen, 1914) | SMF-58534 | female | adult | 3.85 mm | habitus drawings of dorsal and lateral view |
| *N. spicatus* Hodgson, 1910 | SMF-58535 | male | adult | 3.35 mm | habitus drawings of dorsal and lateral view |
| *N. spinosa* (Hodgson, 1902) | SMF-58536 | female | adult | 5.15 mm | habitus drawings of dorsal and lateral view |
| *N. quadrispinosa* (Beddard, 1886) | SMF-58537 | male | adult | 5.15 mm | habitus drawings of dorsal and lateral view |

**Table S1.** Specimens used for taxonomic illustrations shown in Figure 3-11.
